# Evaluation of a Free-Coupon Program for Cervical Cancer Screening Among the Young: A Nationally Funded Program Conducted by a Local Government in Japan

**DOI:** 10.2188/jea.JE20140080

**Published:** 2015-01-05

**Authors:** Yutaka Ueda, Tomotaka Sobue, Akiko Morimoto, Tomomi Egawa-Takata, Chie Hashizume, Hisayo Kishida, Satomi Okamoto, Kiyoshi Yoshino, Masami Fujita, Takayuki Enomoto, Yoshimi Tomine, Jun Fukuyoshi, Tadashi Kimura

**Affiliations:** 1 Department of Obstetrics and Gynecology, Osaka University Graduate School of Medicine, Suita, Osaka, Japan; 1 大阪大学大学院医学系研究科 産科学婦人科学教室; 2 Department of Social and Environmental Medicine, Osaka University Graduate School of Medicine, Suita, Osaka, Japan; 2 大阪大学大学院医学系研究科 環境医学教室; 3 Community Health Division, Central Health Center, Health and Welfare Department, Toyonaka City Hall, Toyonaka, Osaka, Japan; 3 豊中市役所 健康福祉部地域保健課; 4 Department of Obstetrics and Gynecology, Niigata University Graduate School of Medical and Dental Sciences, Niigata, Japan; 4 新潟大学大学院医歯学総合研究科 産科婦人科学教室; 5 Cancer Scan, Tokyo, Japan; 5 キャンサースキャン

**Keywords:** cervical cancer screening, free-coupon, screening rate, consecutive screening

## Abstract

**Background:**

Finding ways to improve the cervical cancer screening rates among young women has been seen as a critical national health problem in many countries, including Japan. The aim of the present study was to evaluate the effects of a free-coupon program for cervical cancer screening conducted by a local government under financial support from the Japanese national government.

**Methods:**

The personal cervical cancer screening information was analyzed for all female residents of Toyonaka City, including any past screening history and clinical results since the year 2009, when a free-coupon program for screening was started. These results were compared to results from 2008, prior to implementation of the free-coupon screening program.

**Results:**

The screening rates of women eligible for the free-coupon peaked dramatically compared to women of similar age who paid for their screening; however, the rates for the ineligible-age population also increased significantly in parallel to those in the free-coupon program, possibly by indirect peer and publicity effects. In women aged 20 to 25 years, the consecutive screening rate after a free-coupon screening was significantly lower than for those women who received a regular residential screening. After a free-coupon screening, the rate for participating in consecutive screenings depended significantly on the institution where the participant received her first screening test.

**Conclusions:**

These results suggest that, for a generation of young women 20–25 years of age, a free-coupon program for cervical cancer screening was effective in increasing the first-time participation rate for screening; however, the increase in first-time participation did not lead to the expected increase in consecutive screenings.

## Abstract (ja)

**背景:**

本邦での子宮がん検診受診率は低く、特に若年女性の受診率の著明な低さは社会問題化している。これに対して2009年度から子宮がん検診受診率向上を目指して無料クーポン事業が開始されたが、当研究はその無料クーポンが検診受診率に与えた影響を評価することを目的とした。

**方法:**

豊中市において、無料クーポン事業が始める前年の2008年度と無料クーポン事業が実施された2009～2012年度における子宮がん検診受診率を年齢別に比較し、また受診者のそれ以前およびそれ以降の子宮がん検診受診状況を解析した。

**結果:**

2009～2012年度の全期間を通じた子宮がん検診受診率は、無料クーポン対象年齢の20歳・25歳・30歳・35歳・40歳でに有意に上昇した。一方、無料クーポンの対象年齢ではない21歳～24歳および26歳～29歳においても受診率は有意に上昇したが、これは20歳・25歳での無料クーポン受診者の継続受診によってもたらされたのではなく、子宮がん検診の認識が広まったことによる初めての検診受診の増加による可能性が示唆された。また、2009年度の20歳・25歳の無料クーポン受診者のその後の継続検診受診率は、同年における通常の住民検診における21歳・26歳の検診受診者のその後の継続検診受診率に比し有意に低率であった。さらに、この20歳・25歳の無料クーポン受診後の継続検診の受診率は、20歳・25歳時の無料クーポンによる検診の受診施設によって有意差があることが判明した。

**結論:**

子宮がん検診無料クーポンは対象年齢および非対象年齢の若年女性の初めての検診受診率を有意に上昇させた。しかし無料クーポンはその後の継続受診には結びついておらず、課題も浮き彫りになった。

## INTRODUCTION

Cancer of the cervix is the second most common cancer in women worldwide, with about 500 000 new cases and 250 000 deaths each year.^1^ Almost 80% of cases occur in low-income countries.^1^ Although a vaccine against the human papillomavirus (HPV) effectively prevents human papillomavirus infection and thus reduces the risk of cervical cancer by around 70%,^2^ about 30% will still develop cervical cancer.

In some countries, including the United States and the United Kingdom, the cervical cancer screening rate is roughly 80%; however, in Japan it is only 25%.^3^ Of particular concern, the screening rate for women aged 20–29 years is less than 10%.^4^ Further, the incidence of cervical cancer among this 20- to 29-year age group has recently been increasing dramatically.^5^ Finding ways to improve the screening rates among this younger generation has been seen as a critical national health problem.

In Japan, it is recommended that women start receiving cervical cancer screening at age 20, to be repeated every 2 years. Even if women skip a screening test in the appropriate second year, they can still undergo a screening test the following year. The local government covers part of the screening costs, and the participant pays the rest, which usually amounts to ¥500 to ¥2000 (approximately $5 to $20 in United States’ dollars [USD]). In 2009, a free-coupon program for screening for cervical and breast cancers was introduced in Japan as a national policy. In this program, a coupon or voucher for a free cervical cancer screening was sent by mail to women aged 20, 25, 30, 35, and 40. The program costs were covered by local governments, with financial support from the national government. Because this free-coupon program was terminated at the end of 2013, all citizens aged 20–44 years in Toyonaka had received a free-coupon only once between 2009 and 2013. A woman aged 20 in 2009, for example, would have received a free-coupon screening in 2009 and undergone a regular screening in 2011 and 2013.

There is an evidence gap as to whether removal of out-of-pocket costs and receipt of an individual invitation letter would be effective for increasing the cervical cancer screening rate, especially in Asia.^6^^–^^9^ However, the reason for this inconsistency is unclear.

Toyonaka is an urban city located in Osaka prefecture. In October 2013, Toyonaka had an area of 38.6 km^2^ and a population of 394 004. Toyonaka is officially acknowledged by the national government of Japan as a core city. In the present study, we evaluated the effectiveness of the free-coupon program in improving cervical cancer screening rates among the younger population of Toyonaka.

It was recently reported that removal of out-of-pocket costs for cervical cancer screening was an effective means of increasing the screening attendance of eligible women.^6^ In the present study, we analyzed for the first time the effects of the free-coupon on the screening rate not only for the eligible women but also for the coupon-ineligible women, as well as the results of the screening tests and the consecutive screening rates following the free-coupon screening.

## MATERIALS AND METHODS

The personal screening information of all female residents aged 20–49 in Toyonaka, including screening history and test results since 2009 (when the registration system was renewed and the free-coupon program was started), was available at an individual level. Only the screening rates aggregated by age groups of 20–24, 25–29, 30–34, 35–39, and 40–44 years were recorded for the year 2008. In Toyonaka, participants in the regular cervical cancer screening program typically paid ¥600 (about $6 USD) for a standard cervical cancer screening.

The rate of cervical cancer screening among the young generation of women (defined here as women aged 20–44 years) for each year between 2009 and 2012 was analyzed. During the period from 2009 to 2012, a free-coupon program was conducted for women at 5-year age intervals, beginning at the recommended starting age of 20 years (ie, ages 20, 25, 30, 35, and 40 years). These screening rates were compared to that of each age group during the index year of 2008, which was just prior to the start of the free-coupon program. A comparison of the rates for those requiring further diagnostic workups and for cancer detections between the free-coupon and regular screening programs was also conducted. The screening histories of the free-coupon group and regular screening program group were analyzed for changes in consecutive screening rates and any links between those rates and the screening sites where the previous screening was performed.

This study was approved by the Institutional Review Board and the Ethics Committee of the Osaka University Hospital.

### Statistical analysis

MedCalc software (MedCalc Software, Mariakerke, Belgium) was used for the statistical analysis. Increases in the screening rate for each age or age group were evaluated by the logistic regression model. Differences in the rates of further diagnostic workups and cancer detection between the free-coupon group and the regular screening group were evaluated using Fisher’s exact test. Differences in consecutive screening rates between a free-coupon group and a regular screening group and between screening sites were also evaluated using Fisher’s exact test. Results were considered to be significant when the *P*-value was less than 0.05.

## RESULTS

### Effect of a free-coupon on young women’s participation in cervical cancer screening

Figure and Table 1 show the yearly rate of cervical cancer screening for 20- to 44-year-old women between the years of 2009 and 2012, when the free-coupon program was being conducted. The screening rates for free-coupon-eligible 20-, 25-, 30-, 35-, and 40-year-old women formed peaks. Compared to screening rates in the year 2008 (prior to the free-coupon program), which were calculated for the age groups of 20–24, 25–29, 30–34, 35–39, and 40–44 years, the screening rates for the 20-, 25-, 30-, 35-, and 40-year-old women exhibited statistically significant increases (rate ratio [RR] 7.1, 95% confidence interval [CI] 5.9–8.6; RR 6.4, 95% CI 5.2–7.1; RR 3.1, 95% CI 2.9–3.3; RR 3.3, 95% CI 3.1–3.5; and RR 3.0, 95% CI 2.8–3.2, respectively; Table 2). The RRs of the 20- and 25-year-olds were especially high, relative to those of the 30-, 35-, and 40-year-olds.

**Figure.**
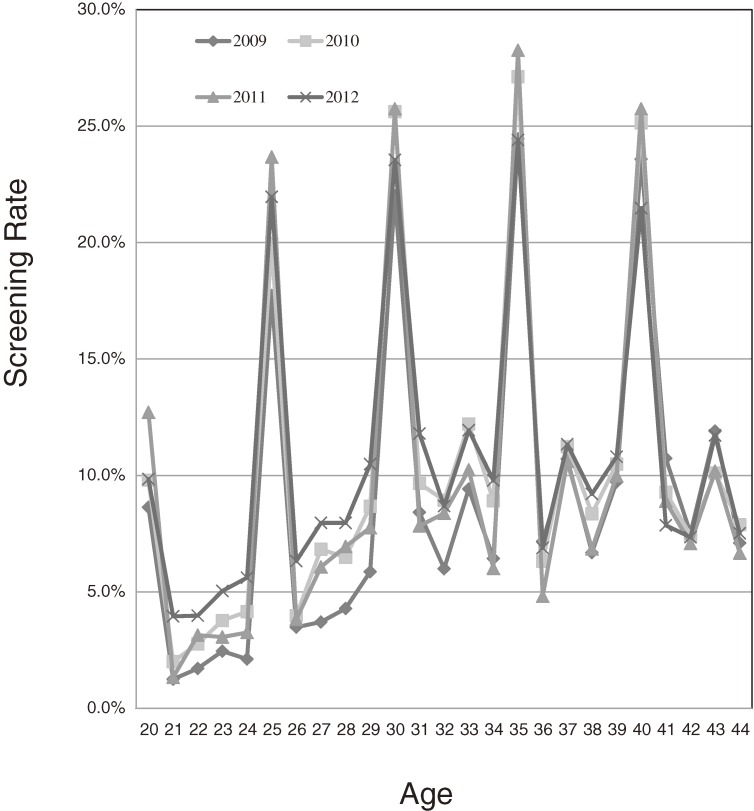
The rate of cervical cancer screening in women 20 to 44 years old in Toyonaka between 2009 and 2012.

**Table 1.**
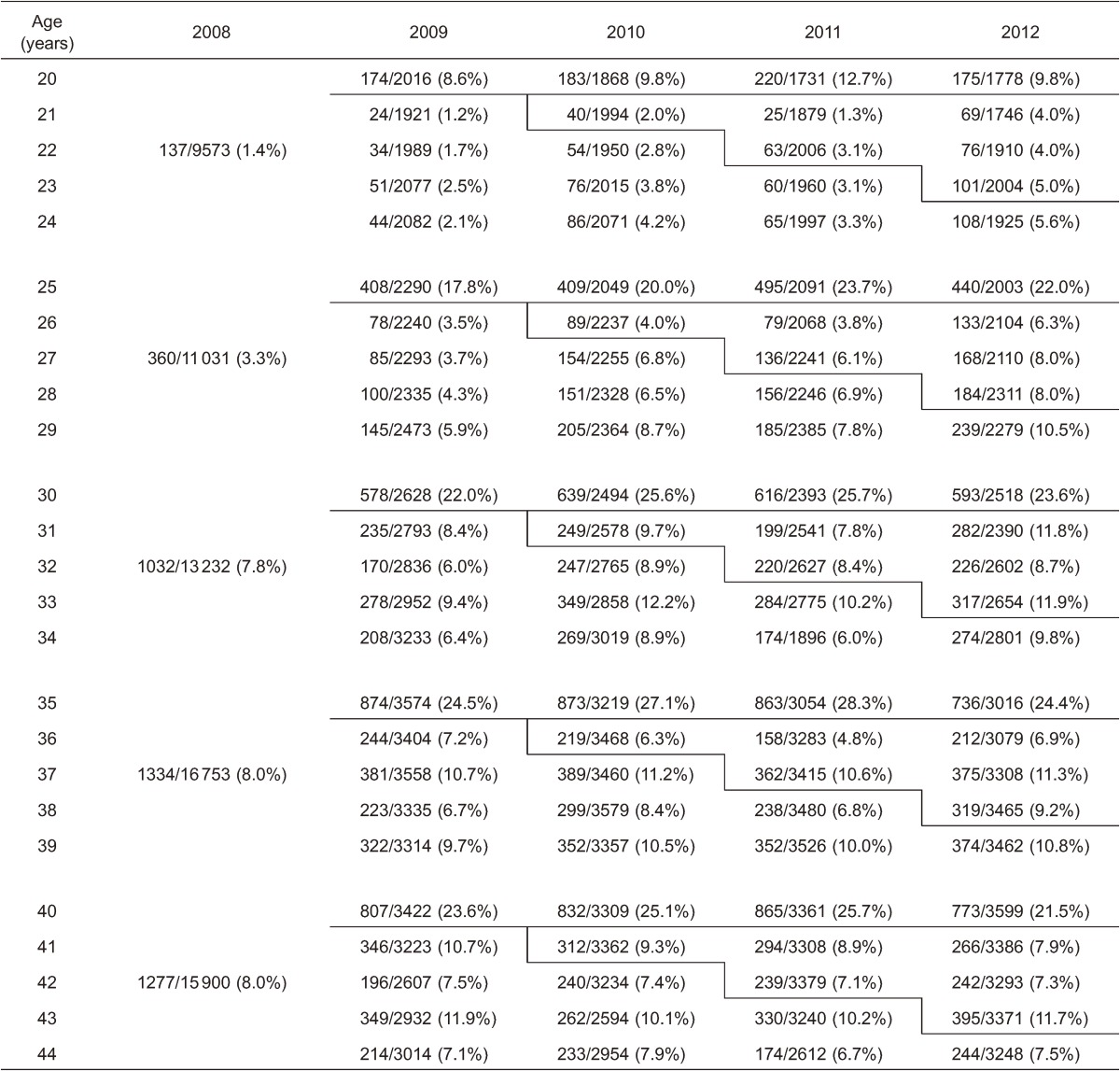
Yearly rate of cervical cancer screening for 20- to 44-year-old women between the years of 2008 and 2012

**Table 2.** Comparison of the cervical cancer screening rate between the index year of 2008 and the free-coupon program years of 2009–2012

Age, years	2008	2009–2012		
	
	Rate of screening	Rate of screening	Rate ratio	95% CI
20	1.4%	10.2%	7.1	5.9–8.6
21–24		3.1%	2.2	1.8–2.6

25	3.3%	20.8%	6.4	5.7–7.1
26–29		6.3%	1.9	1.7–2.2

30	7.8%	24.2%	3.1	2.9–3.3
31–34		9.0%	1.2	1.1–1.2

35	8.0%	26.0%	3.3	3.1–3.5
36–39		8.8%	1.1	1.1–1.2

40	8.0%	23.9%	3.0	2.8–3.2
41–44		8.7%	1.1	1.0–1.2

### Effect of a free-coupon program on participation rates in cervical cancer screening by the ineligible population

Interestingly, the screening rates for the coupon-ineligible population also increased during the study period (Figure). Compared with the screening rate in 2008, the screening rates in the off years from 2009 to 2012 for the coupon-ineligible women in the 21–24, 26–29, 31–34, 36–39, and 41–44 year age groups also significantly increased at the same time that the free-coupon was sent to the eligible 20-, 25-, 30-, 35-, and 40-year-old women (Table 1). The RRs for the 21–24 and 26–29 year age groups were around 2.0 (RR 2.2, 95% CI 1.8–2.6 and RR 1.9, 95% CI 1.7–2.1, respectively); however, those of the 31–34, 36–39, and 41–44 year age groups were around 1.1 (RR 1.2, 95% CI 1.1–1.2; RR 1.1, 95% CI 1.1–1.2; and RR 1.1, 95% CI 1.0–1.2; Table 2).

In order to analyze the reasons for the increased screening rates observed among coupon-ineligible women, the screening history of members of the ineligible population (ie, 21-, 22-, 23-, 24-, 26-, 27-, 28-, and 29-year-old women) post-2009, when the free-coupon program started, who attended screening in 2012 (*n* = 799) was investigated (Table 3). Among 799 women, excluding in-migrants, 531 (66%) had no prior history of screening, while 156 (20%) had a history of an ordinary program screening alone, and 111 (14%) had a history of a free-coupon program screening.

**Table 3.** Past screening history of the population ineligible for a free coupon who received a screening in a regular local program in 2012

Age, years	Number screened (in 2012)	Fixed domicile resident	No history of screening	History of screening with free-coupon	History of screening without free coupon
21	69	65	56 (86%)	9 (14%)	0 (0%)
22	76	65	56 (86%)	9 (14%)	0 (0%)
23	101	79	54 (68%)	19 (24%)	6 (8%)
24	108	90	72 (80%)	0 (0%)	18 (20%)

Subtotal	354	299	237 (80%)	37 (12%)	24 (8%)

26	133	112	81 (72%)	5 (4%)	26 (23%)
27	168	90	28 (31%)	40 (44%)	22 (24%)
28	184	134	88 (66%)	29 (22%)	17 (13%)
29	239	164	97 (59%)	0 (0%)	67 (41%)

Subtotal	724	500	294 (59%)	74 (15%)	132 (26%)

Total	1078	799	531 (66%)	111 (14%)	156 (20%)

### Quality evaluation of cervical cancer screening in a free-coupon program

In order to compare the characteristics of women who received a free-coupon screening and those who were screened in a regular program, the rate of further diagnostic workups and that of cancer detection were analyzed in both groups. The women aged 20, 25, 30, 35, and 40 years were all eligible for a free-coupon, so there were no women among these groups who received a regular program screening and who paid for the costs. The rates of further diagnostic workups and cancer detection during 2009 to 2012 were compared between the women aged 20, 25, 30, 35, and 40 years who received screening with a free-coupon versus those aged 21, 26, 31, 36, and 41 years who received screening in a regular paid program. The rate of requiring further diagnostic workups was 2.0% (240/11 793) in the free-coupon group and 2.3% (80/3553) in the regular program group, indicating no significant difference between the two groups (*P* = 0.43 by Fisher’s exact test). The rate of cancer detection was 8.4 per 100 000 (10/11 793) in the free-coupon group and 8.9 per 100 000 (3/3553) in the regular program group, indicating no significant difference between the two groups (*P* = 1.0 by Fisher’s exact test).

### Rate of consecutive cervical cancer screening after a free-coupon screening

The screening rates of the women aged 20 and 25 years were dramatically increased by the free-coupon program (Figure and Table 1). To assess whether these increased screening rates resulted in increased rates of consecutive screening, the data were analyzed regarding whether or not those women who underwent a free-coupon screening at the ages of 20 or 25 years returned for a subsequent screening. The rate of consecutive cervical cancer screening was compared between the women aged 20 and 25 years who received screening with a free-coupon in the year 2009 and those aged 21 and 26 years who received screening in a regular program in 2009.

In order to investigate the rate of consecutive screening, we excluded from analysis women who out-migrated after a free-coupon screening. In the urban city of Toyonaka, the number of out-migrants was relatively high. Among 582 women aged 20 or 25 years who received a free-coupon screening in the year 2009, 111 persons (19%) moved out of the city within 2 years (Table 4). Among the 102 coupon-ineligible women aged 21 or 26 years who received a screening in a regular program in the year 2009, 20 persons (20%) moved out of the city within 2 years.

**Table 4.** Rates of consecutive cervical cancer screening after a free-coupon screening and a regular screening

	Screening number	Out-migrant within 2 years	Repeated screening within 2 years
Free coupon in 2009			
20 years old	174	19/174 (11%)	10/152^a^ (6.5%)^b^
25 years old	408	92/408 (23%)	40/311^a^ (13%)^c^
Total	582	111/582 (19%)	50/463^a^ (11%)^d^
Regular program in 2009			
21 years old	24	3/24 (13%)	7/21^a^ (33%)^b^
26 years old	78	17/78 (22%)	18/61^a^ (30%)^c^
Total	102	20/102 (20%)	25/82^a^ (30%)^d^

^a^Cases that required further diagnostic workups on initial screening are excluded.

^b,c,d^*P* < 0.001 by Fisher’s exact test.

After excluding the out-migrants, the continuous screening rate was analyzed. In Japan, women aged 20 years or older are invited for cervical cancer screenings at consecutive two-year intervals, with financial support from their local government. The consecutive screening rate of women aged 20 and 25 within the 2-year interval following the introduction of the free-coupon screening program in 2009 was 6.5% for the 20-year-olds (10/152) and 13% for the 25-year-olds (40/311). On the other hand, the rates of re-visits for women aged 21 or 26 years within a similar 2-year period following a screening in the regular program in the year 2009 were significantly higher: 33% for the 21-year-olds (7/21; *P* < 0.001) and 30% for the 26-year-olds (18/61; *P* < 0.001).

When for some reason a person does not receive a screening after a 2-year interval, she can still undergo a screening in the 3rd year with the same financial support. The consecutive screening rate of women aged 20 and 25 within the 3-year interval following the introduction of the free-coupon screening program in 2009 was 16% for the 20-year-olds (24/142) and 22% for the 25-year-olds (63/277; data not shown). On the other hand, the rates of re-visits for women aged 21 or 26 years within a similar 3-year period following screening in the regular program in the year 2009 were significantly higher: 56% for the 21-year-olds (10/18; *P* < 0.001) and 60% for the 26-year-olds (31/52; *P* < 0.001; data not shown).

### Effect of screening site on rate of repeating cervical cancer screening

Next, we investigated the effect of where the screening tests were performed on the consecutive screening rate of women aged 20 or 25 years who received a free-coupon screening and that of those aged 21 or 26 years who received a screening through the regular program in 2009. There were 22 clinics and 6 screening centers where cervical screening test were provided in Toyonaka; however, only 18 of the 22 clinics participated in the 2009 program.

Interestingly, the consecutive screening rates of the 20- and 25-year-olds screened for free at clinic A within the 2-year interval was 25% (22/88), which was significantly higher than the 7% (28/375) reported from the other institutions (*P* < 0.001; Table 5). On the other hand, the consecutive screening rates for 21- and 26-year-olds after a paid screening were slightly (but not significantly) higher at clinic A than at the other screening sites (*P* = 0.11).

**Table 5.** Differences in rates of consecutive screening are related to the screening sites where the previous screening was performed

	Clinic A	Other institutions	*P*-value
Free coupon in 2009			
Subsequent screening within 2 years	22/88 (25%)^b^	28/375^a^ (7%)^b^	<0.001
Ordinary program in 2009			
Subsequent screening within 2 years	13/32 (41%)^c^	12/50^a^ (24%)^c^	0.11

^a^The cases that required further diagnostic workups on initial screening were excluded.

^b^*P* < 0.001 by Fisher’s exact test.

^c^*P* = 0.07 by Fisher’s exact test.

The consecutive screening rates of the 20- and 25-year-olds screened for free at clinic A within the 3-year interval was 46% (37/80), which was significantly higher than the 15% (50/339) reported from the other institutions (*P* < 0.001; data not shown). On the other hand, the consecutive screening rates for 21- and 26-year-olds after a paid screening were slightly (but not significantly) higher at clinic A than the other screening sites (*P* = 0.07).

## DISCUSSION

There is a critical need to improve the rate of cervical cancer screening among younger women in Japan, as well as in many developing countries. The screening rate of women aged 20 to 29 years is still less than 10%,^4^ despite the increasing incidence of cervical cancer in this group.^5^ In addition, due to a media blitz about adverse events following HPV vaccination and a statement by the Ministry of Health, Labor, and Welfare of Japan in June 2013 regarding the suspension of an aggressive recommendation for HPV vaccination, the rate of HPV vaccination has dramatically decreased. Given these situations, the need for improvement in the cervical cancer screening rate among younger women is attracting serious attention. National and local governments therefore enacted a program in which a free cervical screening coupon was sent to 20-, 25-, 30-, 35-, and 40-year-old women to address this problem.

Although many interventions have attempted to remove some of the barriers to cervical cancer screening,^10^^–^^16^ out-of-pocket costs for screening remain a barrier to access in the United States and Japan.^7^ Recently, Tabuchi et al. demonstrated that removal of the out-of-pocket costs by providing a free-screening coupon improved cervical cancer screening participation in Japan.^6^ However, they did not analyze how the screening rate was affected for women who had out-of-pocket costs (because of ineligible age for the free screening). In the present study, the screening rates during 2009 to 2012 were shown to rise sharply among those receiving free screening compared to the rates among those of the same age during the pre-program index year of 2008, especially in the two youngest age groups studied (ie, the women aged 20 or 25; Figure and Table 1). However, the screening rate among coupon-eligible women did not increase significantly between 2009 and 2012 (data not shown). This might imply a limitation of the effect of removal of out-of-pocket costs.

We demonstrated for the first time that the screening rates of the population who were paying for their screening (because they were an ineligible age) also increased significantly during the period of this program. While the rates among coupon-ineligible women did not increase as dramatically as those among coupon-eligible women, there was still a significant improvement over 2008 rates.

Possible reasons for the increased screening rates of the youngest of the free-coupon ineligible population during the free-coupon program might be an return visit for screening in a regular program 1 to 3 years after an initial free-coupon screening, or due to indirect effects of the free-coupon program, including improved education and understanding of cervical (and breast) cancer and enhanced motivation for cancer screening. Peer pressure from family, friends, and colleagues to participate in screening between members of the two groups is also likely.

The rate of repeat screening after receiving a previous free-coupon screening among the women who received a regular screening in 2012 was only 14%. This low rate of repeat screening suggests that the significant increase of screening rates seen among 21- to 24-year-old and 26- to 29-year-old women (RR 2.2 and 1.9, respectively; Table 2) cannot be explained by return visits for a regular screening 1 to 3 years after initial free-coupon screening. The increased screening rates of the ineligible population after the free-coupon program started might be caused by indirect publicity effects of the free-coupon program, including improved understanding of cervical cancer and enhanced motivation for cancer screening in young women (Table 2). This somewhat unexpected effect of the free-coupon program should be confirmed in the future.

It was also demonstrated that the rate of requiring a diagnostic workup and the rate of cancer detection due to the screenings were not markedly different between the free-coupon and paid screening program groups. Perhaps more importantly, it was demonstrated for the first time that the follow-up screening rates were significantly lower in the free-coupon group than in the regular screening group (Table 4). This result shows that the complete removal of out-of pocket costs for cervical cancer screening dramatically inspires young women to attend an initial screening; however, it does not translate to following through for a repeat screening 2 years later. This may be a limitation of the effect of a free-coupon cervical cancer screening program. On the other hand, the women who paid some amount of money for a regular screening program were shown to have a consecutive screening than those who attended a free-coupon screening. These results suggest that the largest problem now is how to inspire women to maintain a regular schedule of subsequent screenings. Understanding why the free-coupon group failed to improve rates of consecutive screening will help in providing a solution.

Interestingly, the consecutive screening rate after a free-coupon screening varied depended on where the participants received their previous screening test. This link to the screening experience may provide a partial explanation for the lack of improvement in consecutive screening rates. In the clinic where the rate of follow-up screening was significantly higher, the doctors and staff had spent enormous time and effort to educate the patient about the importance of the screening test to detect cervical cancer; however, it is difficult to statistically compare these educational efforts with those of other institutions. Education is but a part of the screening experience. Institutional reputation, location, scheduling convenience, and waiting room and screening room ambiance all play a role in whether the patient perceives the screening experience as worth repeating. These features of the screening experience are all difficult to quantify and compare statistically.

The Community Preventive Services Task Force demonstrated effectiveness of removal of out-of-pocket costs for breast cancer screening in increasing screening rates for breast cancer; however, evidence with respect to improving cervical cancer screening rates was insufficient.^17^ The present study provided some evidence that a free-coupon program is also effective in improving cervical cancer screening rates.

In the present study, the effects of a free-coupon program on the screening rate of both eligible and ineligible women, the rates of requiring further diagnostic workups and cancer detection of a free-coupon screening, and the consecutive screening rate following a free-coupon screening in Toyonaka were analyzed. However, data from only one urban city were analyzed, which is a limitation of the present study. A larger, nation-wide study is necessary to confirm our findings.

## ONLINE ONLY MATERIAL

Abstract in Japanese.
