# Supplementary material for: Evaluation of a Free-Coupon Program for Cervical Cancer Screening Among the Young: A Nationally Funded Program Conducted by a Local Government in Japan
Source: J Epidemiol. 2015 Jan 5;25(1):50–6. doi: 10.2188/jea.JE20140080 (PMC4275438; doi:10.2188/jea.JE20140080)
Supplement: Abstract in Japanese. [file je-25-050-s001.pdf]

本邦における若年女性に対する子宮がん検診無料クーポン事業の評価

上田　豊[1)]、祖父江友孝[2)]、森本晶子[1)]、高田友美[1)]、橋爪千恵[3)]、岸田久世[3)]、岡本里美[3)]、吉野　潔[1)]、藤田征巳[1)]、榎本隆之[4)]、遠峰良美[5)]、福吉潤[5)]、木村　正[1)]

1) 大阪大学大学院医学系研究科　産科学婦人科学教室

2）大阪大学大学院医学系研究科　環境医学教室

3）豊中市役所　健康福祉部地域保健課

4）新潟大学大学院医歯学総合研究科　産科婦人科学教室

5）キャンサースキャン

背景

本邦での子宮がん検診受診率は低く、特に若年女性の受診率の著明な低さは社会問題化している。これに対して 2009 年度から子宮がん検診受診率向上を目指して無料クーポン事業が開始されたが、当研究はその無料クーポンが検診受診率に与えた影響を評価することを目的とした。

方法

豊中市において、無料クーポン事業が始める前年の 2008 年度と無料クーポン事業が実施された 2009～2012 年度における子宮がん検診受診率を年齢別に比較し、また受診者のそれ以前およびそれ以降の子宮がん検診受診状況を解析した。

結果

2009～2012 年度の全期間を通じた子宮がん検診受診率は、無料クーポン対象年齢の 20 歳・25 歳・30 歳・35 歳・40 歳でに有意に上昇した。一方、無料クーポンの対象年齢ではない 21 歳～24 歳および 26 歳～29 歳においても受診率は有意に上昇したが、これは 20 歳・25 歳での無料クーポン受診者の継続受診によってもたらされたのではなく、子宮がん検診の認識が広まったことによる初めての検診受診の増加による可能性が示唆された。また、2009 年度の 20 歳・25 歳の無料クーポン受診者のその後の継続検診受診率は、同年における通常の住民検診における 21 歳・26 歳の検診受診者のその後の継続検診受診率に比し有意に低率であった。さらに、この 20 歳・25 歳の無料クーポン受診後の継続検診の受診率は、20 歳・25 歳時の無料クーポンによる検診の受診施設によって有意差があることが判明した。

結論:

子宮がん検診無料クーポンは対象年齢および非対象年齢の若年女性の初めての検診受診率を有意に上昇させた。しかし無料クーポンはその後の継続受診には結びついておらず、課題も浮き彫りになった。

キーワード：子宮がん検診、無料クーポン、受診率、継続検診
